# Age-related differences in visual confidence are driven by individual differences in cognitive control capacities

**DOI:** 10.1038/s41598-022-09939-7

**Published:** 2022-04-10

**Authors:** Lena Klever, Pascal Mamassian, Jutta Billino

**Affiliations:** 1 Experimental Psychology, Justus Liebig University Giessen, Giessen, Germany; 2 Center for Mind, Brain, and Behavior (CMBB), University of Marburg and Justus Liebig University Giessen, Giessen, Germany; 3 Laboratoire des Systèmes Perceptifs (CNRS UMR 8248), Département d’Études Cognitives, École Normale Supérieure, PSL University, 75005 Paris, France

**Keywords:** Psychology, Human behaviour

## Abstract

Visual perception is not only shaped by sensitivity but also by confidence, i.e., the ability to estimate the accuracy of a visual decision. Younger observers have been reported to have access to a reliable measure of their own uncertainty when making visual decisions. This metacognitive ability might be challenged during ageing due to increasing sensory noise and decreasing cognitive control resources. We investigated age effects on visual confidence using a visual contrast discrimination task and a confidence forced-choice paradigm. Younger adults (19–38 years) showed significantly lower discrimination thresholds than older adults (60–78 years). To focus on confidence sensitivity above and beyond differences in discrimination performance, we estimated confidence efficiency that reflects the ability to distinguish good from bad perceptual decisions. Confidence efficiency was estimated by comparing thresholds obtained from all trials and trials that were judged with relatively higher confidence, respectively. In both age groups, high confidence judgments were associated with better visual performance, but confidence efficiency was reduced in older adults. However, we observed substantial variability across all participants. Controlling for age group, confidence effciency was closely linked to individual differences in cognitive control capacities. Our findings provide evidence for age-related differences in confidence efficiency that present a specific challenge to perceptual performance in old age. We propose that these differences are driven by cognitive control capacities, supporting their crucial role for metacognitive efficiency.

## Introduction

Human behaviour and its underlying neural mechanisms are mostly studied with a specific focus on a particular functional domain, e.g., perception, cognition, motivation, or motor functions. Although this approach has allowed detailed models and theories, complexity of behaviour can only be captured comprehensively when interactions across domains are also considered^1–3^. A particularly influential, well-investigated higher-level concept that shapes behaviour is metacognition. It refers to the ability to evaluate the quality and consequences of one’s own thoughts and behaviours^4,5^. Metacognition has been widely studied in psychology during the last decade (for review, see^6^). There is consensus that it is key for optimizing performance by balancing actual outcome and subjective estimates of its quality. However, a better understanding of individual differences in metacognitive resources and their impact on behavioural performance is just beginning to emerge.

Individual differences are particularly pronounced in the ageing population, offering a unique window to possible variability in metacognitive efficiency. Ageing, from a behavioural perspective, can be understood as an umbrella term that incorporates gradually changing resources in all functional domains and at the same time adaptive mechanisms that can stabilize performance. Although the view of ageing as a process of deterioration and decline might still be prominent, understanding of age-related differences has gradually shifted towards a more complex characterization, including stability, decline, and compensation^7–9^. Metacognition could crucially contribute to optimizing performance in the face of age-related resource decline^10–13^. However, evidence so far has remained equivocal.

Since the prefrontal cortex has been consistently identified as a critical neural functional correlate of metacognition^14–16^, vulnerabilities during ageing have been assumed. Prefrontal areas are subject to the most pronounced age-related volume loss^17,18^. In addition, consistent with the involvement of the prefrontal cortex, metacognition is considered to be closely related to higher-order cognitive processes, i.e., executive function^19,20^. Executive function is not unitary but involves a number of components that have been vividly debated over time^21,22^. There is however consensus on three functional core components, namely updating, shifting, and inhibition^23,24^, that crucially fuel adaptive information processing and thereby efficient behavioral control. Age-related decline in executive function, indeed, is the most prominent facet of cognitive ageing^25–27^. Thus, clear predictions about age-related effects on metacognition can be derived, though it still seems a matter of debate how sensitive this functional capacity is to age.

The majority of studies that have investigated age-related differences in metacognition so far has focused on memory performance, so called metamemory^28^. Metamemory is typically assessed by subjective measures of how confident an individual feels about the quality of their own memory performance, e.g., by giving a prospective or retrospective judgement on a rating scale. Several studies have reported an increased mismatch between actual performance and the judgements on one’s own abilities in older adults^29–36^. They tend to be overconfident about the quality of their memory performance. On the other hand, there are almost as many studies that have found only minor or even no age effects on the accuracy of metamemory^28,37–39^. Metacognition in other functional domains, e.g., problem solving, linguistics, perception, even seems to elude any age effects^28,40,41^. Heterogenous results might be due to the use of rating scales for assessing confidence. Ratings could confound individual biases to distribute judgements across the scale, so evaluation of metacognition sensitivity from ratings is challenging^42,43^. Moreover, confidence judgements in commonly used cognitive tasks are made on rather complex decisions involving multiple criteria that might generate additional biases hard to control.

Given these issues, the investigation of metacognition in perceptual tasks has attracted increasing consideration, establishing the term metaperception as a subtype of metacognition (for review, see^42^). Perceptual tasks qualify for a well-structured assessment of metacognition since they typically are characterized by simple decisions based on some sensory evidence, e.g., contrast or orientation discrimination. Metacognition in a perceptual task describes an observer’s ability to monitor, evaluate, and control their own perception. Perceptual confidence provides a prototypical example for this ability. Perceptual decisions are accompanied by a subjective sense of (un)certainty, depending on the strength of sensory signals. Having access to a reliable measure of one’s own uncertainty is a crucial aspect of perceptual confidence. Confidence about one’s own decisions is fundamentally related to the accuracy of decisions (e.g.,^44^, see also^45^). Observers will report high confidence when their perceptual decision is objectively correct, and low confidence when it is objectively incorrect. During ageing the quality of confidence judgements in perceptual tasks, i.e., how well they map the correctness of decisions, might be particularly challenged by pronounced age-related sensory decline due to peripheral vulnerabilities and increasing noise in neural representations^10,46–48^ that hamper the evaluation of (un)certainty.

Only a single study so far has considered age effects on metacognition in a perceptual task. Palmer and colleagues^28^ assessed metacognition in the memory as well as in the visual domain, studying a sample that covered the adult age range from early to late adulthood. Though providing first insights into age-related decline in metacognitive efficiency across functional domains, some conclusions appear unsettled because of several ambiguities in the results. Metacognitive efficiency did not decline consistently across perception and memory. While metacognition in the perceptual task decreased with age, only minor differences were found in the memory task. Given that evidence for domain-general versus domain-specific metacognitive systems remains controversial^49–52^, the result might point to critical confounds inherent to the chosen tasks. In addition, the reported dissociation between metacognitive efficiency and executive function awaits scrutiny since the latter was assessed rather rudimentarily by a single measure, putatively not capturing capacities comprehensively.

We aimed to investigate how age affects metacognitive abilities in visual perception using a confidence forced-choice paradigm^42,53^. In this paradigm, observers are asked for two perceptual decisions sequentially, e.g., in our study on two contrast discrimination tasks, and then have to indicate about which of the two decisions the feel more confident. This method allows to assess perceptual performance precisely and to derive a bias-free measure of confidence, avoiding confounds that could emerge from confidence rating scales. Confidence measures in this paradigm are not affected by possible idiosyncratic confidence biases that have been reported in older adults^29,32^. It allows analyses based on the signal detection theory framework, controlling for differences in perceptual task performance. The procedure also provides the opportunity to analyse response times that change significantly during ageing and could affect the calibration of confidence judgements in perceptual tasks^54,55^. Furthermore, we considered executive function as a cognitive key capacity that might play a critical role for confidence efficiency. We hypothesized that older adults show decreased metacognitive abilities in perceptual tasks and that these age effects are crucially driven by individual differences in cognitive control capacities, i.e., executive function.

## Methods

### Participants

A total of 30 younger adults (18 females) and 30 older adults (17 females) participated in this study. The participants' age ranged from 19 to 38 years with a mean of 24.6 years (*SD* = 4.4) in the younger group and from 60 to 78 years with a mean of 68.8 years (*SD* = 4.7) in the older group. Recruitment of participants was managed by calls for participation at the University of Giessen and in local newspapers. Older adults reported slightly fewer years of school education than younger adults, 12.1 years (*SD* = 1.5) and 12.9 years (*SD* = 0.5), respectively. Higher academic degrees were completed by 66.7% of older adults. All younger adults either were currently enrolled in an academic program or had already completed a degree (43.3%). Our sample thereby is characterized by a bias towards higher educational levels when compared with the basic population. Higher educational attainment has been discussed to slow down age-related changes so that an underestimation of age-related differences in our given sample might be considered (^56^, but see^57^). However, most importantly, educational background is comparable across both age groups, avoiding a potential confound with regard to the planned comparisons. Any history of ophthalmologic, neurologic, or psychiatric disorders as well as medications presumed to interfere with visual functioning were screened out by a detailed interview protocol. Older adults were further screened with regard to visual acuity and mild cognitive impairment. We measured visual acuity binocularly using the Freiburg Visual Acuity Test^58^ and confirmed normal or corrected-to-normal acuity, i.e., decimal acuity > 0.7. In addition, we applied the Montreal Cognitive Assessment Scale using a cut-off score of ≥ 23, excluding pathological cognitive decline^59–61^. Table 1 gives an overview of the main characteristics of participants. Methods and procedures were approved by the local ethics committee at Justus Liebig University Giessen and adhered to the principles of the Declaration of Helsinki^62^. All participants provided informed written consent prior to the experiment. Participants were compensated with course credits or money.

**Table 1:** Characteristics of participants and cognitive results.

	Older adults (*n* = 30)		Younger adults (*n* = 30)	
	*M* (*SD*)	Range	*M* (*SD*)	Range
Age (years)	68.8 (4.7)	60–78	24.6 (4.4)	19–38
School education (years)	12.1 (1.5)	9–13	12.9 (0.5)	10–13
MoCA (raw score)	27.7 (1.6)	24–30	*n.a.*	*n.a.*
DSST (raw score)	60.2 (11.3)	42–99	82.3 (11.8)	59–106
TMT-B (s)	77.4 (23.0)	39.3–133.2	43.5 (13.2)	24.2–89.4
VST-C (s)	69.1 (42.2)	21.9–193.9	25.5 (5.4)	15.5–36.0
LPS-3 (raw score)	16.9 (3.4)	8–22	22.1 (3.5)	16–31
Digit span (max. backwards)	4.4 (0.9)	3–7	5.0 (1.0)	4–7

*MoCA* Montreal Cognitive Assessment, *DSST* Digit Symbol Substitution Test, WAIS-IV; *TMT-B*, Trail Making Test, part B, *VST-C* Victoria Stroop Test colour naming, *LPS-3* LPS intelligence scale, subtest 3, logical reasoning; *M*: mean; *SD*: standard deviation; *n.a.* not assessed; where applicable, comparisons between older and younger adults using *t*-tests yielded significant differences in the reported measures, all *p’*s ≤ 0.01.

### Assessment of individual differences in cognitive abilities

We characterized cognitive abilities of our participants using a battery of established measures that particularly allowed for evaluation of executive function (EF). Table 1 summarizes participants’ performance in the different cognitive tasks. We aimed to assess EF comprehensively, considering key facets of cognitive control processes^23^. It is important to note that so far metacognition has not been linked to a specific candidate EF facet^19,20^. Thus, our assessment was tailored for covering the EF concept broadly and deriving a composite measure that provides a robust indicator of cognitive control capacities that are supposed to support efficient information processing. Critical single measures included: the Digit Symbol Substitution Test (DSST)^63^, measuring updating ability; the Trail Making Test part B (TMT-B)^64^, measuring shifting ability; the Victoria Stroop Test colour naming (VST-C)^65,66^, measuring inhibition ability; the LPS-3^67^, a subtest of a major German intelligence test battery, measuring nonverbal reasoning ability. In order to combine the single measures, we consistently scaled them so that higher scores indicated better cognitive control capacities. Then, for each participant a global EF score was derived by averaging the *z*-scores obtained for the individual measures. In addition, we assessed the maximal backward digit span^68^ in order to evaluate short-term memory capacity that qualified as a possible confounding issue given the procedural details of our task procedures.

### Setup and stimuli

Visual stimuli were presented on a calibrated 32-inch Display++ LCD monitor (Cambridge Research Systems, Rochester, UK) with a spatial resolution of 1920 × 1080 pixels and a refresh rate of 120 Hz noninterlaced. The setup was placed in a darkened room and participants were seated at a distance of 100 cm in front of the monitor, resulting in a display size of 41° × 23°. White and black pixels had a luminance of 112.7 and 0.1 cd/m^2^, respectively, measured with a CS-2000 Spectroradiometer (Konica Minolta). Stimulus presentation was controlled by MATLAB using the Psychophysics toolbox^69,70^. A standard gamepad was used as input device (Microsoft SideWinder).

Stimuli were vertical Gabor patches displayed on an average grey background. Sinusoidal gratings had a spatial frequency of 0.8 cyc/° with randomized phase and the standard deviation of the Gaussian envelope was 1°. The contrast of the Gabor patches was sampled from seven different levels ranging from 13 to 31% in steps of 3%. The stimulus configuration consisted of two Gabor patches presented to the left and right of a central fixation dot at 4.2° eccentricity along the horizontal meridian. The fixation dot was black and had a diameter of 0.2°. One Gabor patch, i.e., the standard patch, had a fixed contrast of 22%, whereas the contrast of the other Gabor patch, i.e., the test patch, varied. Laterality of standard and test patches, respectively, was randomized.

### Procedure

**Figure 1:**
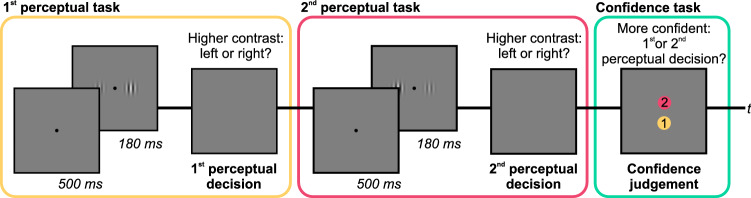
Trial procedure of the confidence forced-choice paradigm. Participants were presented with two consecutive perceptual tasks in which they had to decide which of two simultaneously presented Gabor patches appeared higher in contrast. After the second perceptual decision, they were asked for a confidence judgement, i.e. they had to indicate which of the two perceptual decisions they felt more confident about. Please note that colour is here used to illustrate the consecutive steps in each trial and was not used in the actual procedure.

We assessed metacognitive abilities in visual perception using a confidence forced-choice paradigm^53,71,72^. Figure 1 depicts a typical trial.

Each trial consisted of two consecutive perceptual tasks, specifically contrast discrimination tasks, and a final confidence task. A fixation dot was shown for 500 ms, which was followed by two Gabor patches presented simultaneously for 180 ms. Then the display turned grey and participants decided whether the left or right patch appeared higher in contrast (first perceptual decision). Responses were entered with the respective index fingers using the trigger buttons on the back of the gamepad. Then, an equivalent second task followed, using different patches and another contrast decision was made (second perceptual decision). Afterwards, participants indicated which of the two perceptual decisions they felt more confident about (confidence judgement). The response was given with the right thumb using two vertically aligned buttons on the top side of the gamepad. The buttons were mapped to the first or second perceptual decision, respectively. The mapping was visualized on the display and balanced across participants.

Before data collection, a detailed instruction protocol and sufficient practice trials secured that participants were familiar with the stimulus configuration, could comfortably follow the trial procedure, and handled the gamepad effortlessly. Subsequently, participants completed a total of 420 trials, subdivided into 6 blocks with 70 trials each. The number of trials was determined as a compromise between a sufficiently large number to properly estimate confidence and a session duration sufficiently short to reduce fatigue. Contrast levels of the test patches in the two consecutive contrast discrimination tasks were independently varied according to the method of constant stimuli, i.e., each of the 7 contrast levels was presented in 60 trials for the first and second contrast discrimination task, respectively.

### Data analyses

Based on participants' confidence judgements, we divided perceptual decisions into two confidence sets: The first set included perceptual decisions that were chosen in the confidence task, i.e., they were associated with a relatively higher confidence, and this set was therefore labelled as *chosen*. The second set considered the ensemble of all perceptual decisions and was labelled as *unsorted*. We analysed perceptual performance for both sets by fitting cumulative Gaussian functions to the percentage of responses in which observers reported the contrast of the test patch as higher than the standard patch. The inverse standard deviation of these functions is a measure of contrast sensitivity. We used the psignifit 4 toolbox in Matlab that provides an accurate Bayesian estimation of psychometric functions and has been shown to be robust to overdispersion in measured data^73^. Goodness of fit of the psychometric functions was assessed with the measure of deviance *D* which supported good fits between the model and the data. Both sets showed similar Goodness of fit measures (*t*(58) = 1.82, *p* = 0.074, 95% CI [− 0.117, 2.506], *d* = 0.26).

We quantified metacognitive efficiency, i.e., the relative sensitivity gain driven by confidence, calculating a confidence modulation index (CMI) according to Eq. (1). The CMI gives the sensitivity increase for the set of decisions chosen as confident relative to the set of unsorted decisions as a percentage of the sensitivity derived from the unsorted decisions.

1 $$CMI=100\times \frac{{Sensitivity}_{chosen}-{Sensitivity}_{unsorted}}{{Sensitivity}_{unsorted}}$$

An individual observer who derives their confidence judgements completely dissociated from their perceptual decisions will show a CMI close to zero. However, the closer the confidence judgement is linked to the actual accuracy of the perceptual decision, the higher the CMI will be, indicating better metacognitive sensitivity. Given that the CMI provides a proportional measure, values were arcsine-square-root transformed before they were submitted to statistical procedures. Inspecting the distribution of CMIs in our sample, we identified outlier data for one older participant. Their CMI deviated more than 1.5 times the interquartile range from the range borders of the complete sample. In order to enhance validity of our data and reduce unsystematic noise, we discarded this participant from our analyses.

Processing time measures for perceptual decisions were explored using median response times (RT). Response times below 100 ms and larger than 3000 ms were discarded because they were considered as anticipatory or delayed, respectively. The exclusion rate was less than 1% for each participant. Since perceptual decision times vary with stimulus intensity and confidence in a given task^54,55^, we disentangled both parameters by using a model introduced in previous studies^74,75^. The model separates the effects of stimulus intensity and confidence on response times, allowing for a specific evaluation of both factors. We first normalized stimulus values for each individual considering their psychometric functions. We calculated the signed distances *S* between the 7 used stimulus intensities and the point of subjective equality in standard deviation units of the psychometric function. Chosen and unsorted confidence sets were considered separately. We then fitted an exponential model with three free parameters to the median RTs for each of the 7 stimulus intensity levels. The model is defined by Eq. (2). *RT*(*S*) gives the fitted RT for a normalized stimulus intensity level *S*. *C* gives the corresponding mean confidence across all included perceptual decisions. We encoded confidence with 1 for perceptual decisions that were selected in the confidence choice task and with 0 for perceptual decisions that were not chosen.

2 $$RT\left(S\right)=\alpha - \beta { e}^{-\frac{1}{2}{S}^{2}}- \gamma C$$

The model yields three parameters, i.e., α, giving the generic RT, β, capturing the exponential change in RT due to differences in stimulus intensity, and γ, capturing the linear change in RT due to confidence.

Sensitivity and RT data were analysed by mixed ANOVAs with the within-subject factor *confidence set* (chosen vs. unsorted) and the between subject factor *age group* (older adults vs. younger adults). *T*-tests were used for age group comparisons of the CMI, cognitive measures, and RT parameters. If Levene’s test indicated unequal variances, degrees of freedom were adjusted appropriately. Associations between CMI and critical parameters were investigated by correlational analyses. For group comparisons and correlational analyses, we computed 95% percentile confidence intervals using 2000 bootstrap samples. A significance level of α = 0.05 was applied for all statistical analyses and tests were two-sided. If not stated otherwise, descriptive values are given as means ± 1 SEM.

## Results

We initially explored the overall response patterns of older and younger adults in the confidence forced-choice paradigm. Age effects on visual confidence were then analysed in detail by exploiting contrast sensitivity functions derived from the chosen und unsorted confidence sets, respectively. Differences in metacognitive efficiency were scrutinized considering the role of processing speed and executive functions.

### Overview of response patterns

**Figure 2:**
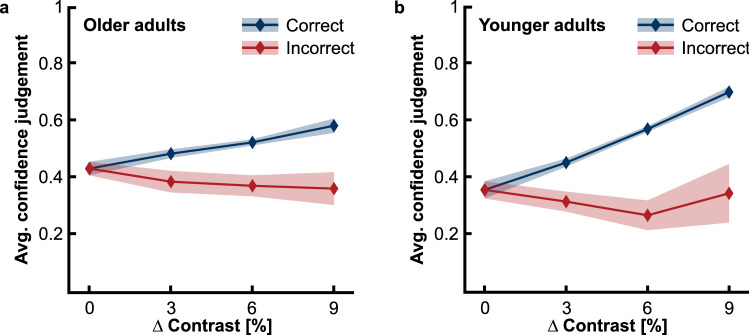
Average confidence judgements for perceptual decisions at different task difficulty levels, plotted separately for correct and incorrect decisions. (**a**) Data for older adults. (**b**) Data for younger adults. Task difficulty level is given as absolute contrast difference between the standard and test patches; task difficulty decreases with difference. Confidence judgements were coded as 1 for chosen and as 0 for not chosen. Please note that confidence judgements were made between two perceptual tasks in a trial. The probability of choosing a decision as confident depends on the difficulty of the other task. We collapsed confidence judgements across the different difficulties. The correctness label for perceptual decisions on patch pairs with zero contrast difference is arbitrary. Shaded areas give 95% confidence intervals.

Figure 2 illustrates confidence judgements for perceptual decisions at different task difficulty levels, i.e., different contrast differences between the standard and test Gabor patches. The separation of data for correct and incorrect decisions provides a rough overview of visual confidence in our paradigm.

In general, participants more often felt confident about their perceptual decisions when these were objectively correct than incorrect, indicating that they evaluated their performance appropriately. This difference in average confidence judgements for correct and incorrect decisions increased when task difficulty decreased. The data patterns hence support that our paradigm captured metacognitive abilities in visual perception in both age groups. However, Fig. 2 also suggests age-related differences since the separation of data for correct and incorrect decisions is clearly less pronounced in older adults.

**Figure 3:**
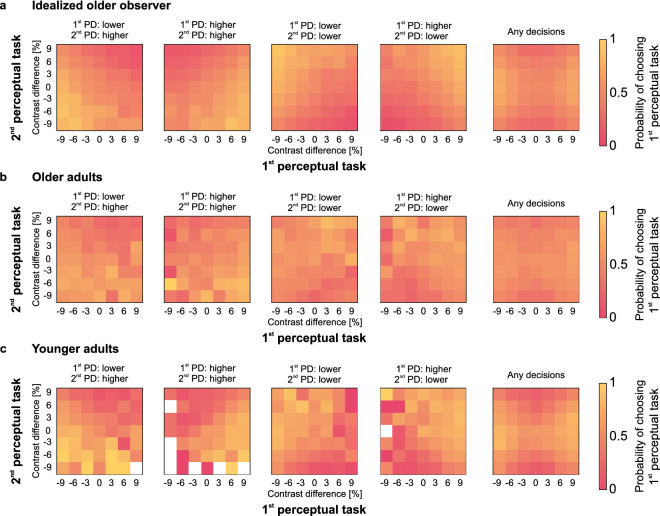
Descriptive illustration of metacognitive abilities in visual perception giving (**a**) an idealized older observer simulated to have a sensory noise equal to the average of the older adults, (**b**) older adults, and (**c**) younger adults. The first four plots in each panel show the probability of choosing the 1st perceptual decision (PD) as the most likely to be correct in the confidence judgement, i.e., associating it with relatively higher confidence, for each of the four possible combinations of perceptual decisions in the two consecutive contrast discrimination tasks. Decisions here apply to the test patches, i.e., code whether the test patches were indicated as lower or higher in contrast than the standard. The last plots on the right show the probability across all trials. The *x*- and *y*-axes give the contrast difference between the test patches and the standard patch in the first and second perceptual tasks, respectively. Metacognitive ability is indicated in these plots by a pattern of probabilities that dynamically depends on task difficulty, i.e., absolute contrast difference, and correctness of the perceptual decisions in both consecutive tasks. White cells in these plots represent the specific combination of consecutive perceptual decisions and stimulus strengths that did not occur in our data set.

A more detailed description of the confidence judgement patterns in older and younger adults is given in Fig. 3, showing all pairs of stimulus difficulties that were subjected to a confidence choice. For comparison, the figure also shows a simulated idealized observer that makes its confidence judgments as well as one would predict from the sensory noise that controls perceptual performance. Here, sensory noise was chosen as the average for the older adults. The probabilities of choosing the first perceptual task as more confident are shown separately for each task difficulty and each combination of perceptual decisions, respectively. The panel in the last column is an aggregate of all four possible pairs of perceptual decisions. Metacognitive abilities are reflected in each map by a pattern of probabilities that varies in two dimensions. Probabilities of choosing the first perceptual task should gradually increase with contrast difference values in the first perceptual decision. In parallel, they should gradually decrease with contrast difference values in the second perceptual decision. The simulated idealized observer pattern demonstrates that sensory noise in older adults and the chosen stimulus difficulties are suitable to expect an appropriate range of confidence judgements. It also provides a critical reference for evaluating the empirical patterns. Confidence probability maps for both age groups reflect metacognitive abilities in the perceptual task. However, the expected patterns are prominent in younger adults, whereas in older adults the gradient of probabilities is substantially blurred. Importantly, the aggregated patterns appear symmetric, anchored at minimal stimulus strengths, ruling out critical response biases due to task order.

In summary, the exploration of response patterns in the confidence forced-choice paradigm suggests that in both age groups participants appropriately derived confidence judgements on their perceptual decisions and thus demonstrated metacognitive abilities. However, evidence for age-related differences emerges and is followed up by quantifying how close confidence judgements are linked to perceptual decisions.

### Psychometric analyses

**Figure 4:**
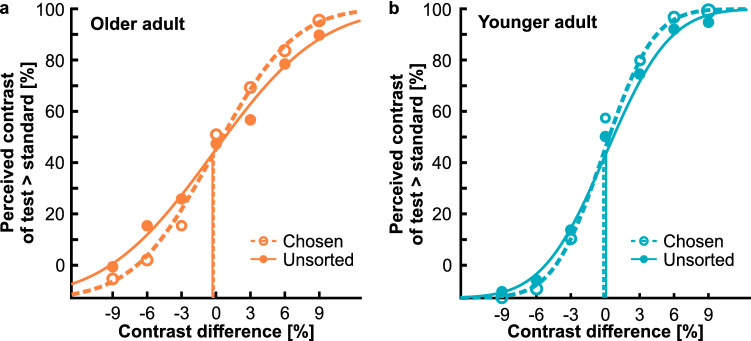
Psychometric functions of contrast discrimination for (**a**) an example older adult and (**b**) an example younger adult. Proportion of decisions indicating higher contrast of the test patch compared to the standard patch is plotted as function of stimulus intensity given as the contrast difference between the test patch and the standard patch. Dashed lines and open dots represent data from the chosen confidence set, solid lines and closed dots represent data from the unsorted confidence set.

We were initially interested in determining whether contrast sensitivity varies between the two confidence sets, i.e., chosen and unsorted sets, and between the groups of older and younger adults. We consistently observed higher contrast sensitivity for the chosen confidence set than for the unsorted confidence set, a signature of metacognitive sensitivity. Figure 4 shows example psychometric functions of contrast discrimination for a representative older (a) and younger (b) adult, respectively. The functions derived from the two confidence sets differ in slope, indicating higher contrast sensitivity for the chosen confidence set. Points of subjective equality lie close to each other.

**Figure 5:**
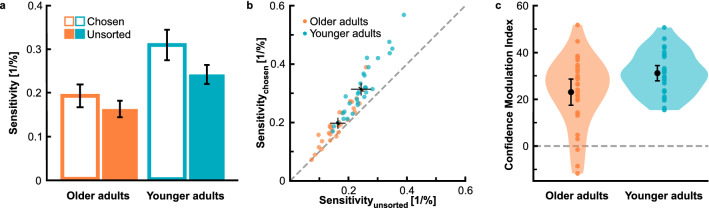
Contrast sensitivity and confidence. (**a**) Average contrast sensitivity as a function of age group and confidence set; open bars illustrate data from the chosen confidence set, closed bars represent data from the unsorted confidence set. (**b**) Contrast sensitivity for the chosen confidence set plotted against contrast sensitivity for the unsorted confidence set; each dot represents data from an individual participant, data for older and younger adults are plotted in different colours; dashed line marks the identity line; black closed dots give average sensitivities in each age group. (**c**) Confidence Modulation Index (CMI) as a function of age group; CMIs give the percental sensitivity increase from the set of unsorted trials to the set of chosen trials; coloured dots illustrate individual data and black dots represent the mean; shaded areas display 95% of the data distribution smoothed by a kernel density function. Error bars give 95% confidence intervals.

Analysis of pooled sensitivity data corroborated inspection of the example psychometric functions. Figure 5a illustrates contrast sensitivity we determined for each confidence set in both age groups. We submitted sensitivity data to a two-factorial ANOVA with *age group* as between-subjects factor and repeated measures on the factor *confidence set*. The analysis yielded significant main effects of *age group*, *F*(1, 57) = 30.30, *p* < 0.001, η_p_^2^ = 0.35, and *confidence set*, *F*(1, 57) = 114.79, *p* < 0.001, η_p_^2^ = 0.67. However, these main effects were qualified by a significant interaction between both factors, *F*(1, 57) = 14.67, *p* < 0.001, η_p_^2^ = 0.21. The interaction effect was followed up by *t*-tests. They corroborated lower sensitivities in older adults for both confidence sets (both *p*’s < 0.001). Effect sizes were similar, i.e., *d* = 1.39 for the chosen and *d* = 1.44 for the unsorted confidence set. The sensitivity advantage for the chosen confidence set was significant for both age groups (both *p*’s < 0.001); however, the difference was less pronounced in older adults, i.e., *d* = 0.42 vs. *d* = 0.55, respectively.

Figure 5b highlights these findings by giving a scatterplot of sensitivities for the unsorted confidence set against sensitivities for the chosen confidence set. Data for older and younger adults are illustrated in different colours. Whereas individual data points for younger adults lie exclusively above the diagonal identity line, those for older adults overall lie closer to and sometimes even marginally below it. Average values show not only lower sensitivities but also a smaller shift from the identity line in older adults. Confidence intervals suggest similar data precision in both age groups.

We further inspected whether the points of subjective equality (PSE) differ between the chosen and unsorted confidence sets. PSEs should logically lie close to zero, i.e., standard and test patches should be indistinguishable when there is no contrast difference. A shift of PSEs for the chosen confidence set could indicate that confidence judgements rely on a biased criterion and thus metacognitive efficiency is inherently limited. Comparisons of PSEs for the chosen and unsorted confidence sets yielded consistent results. For older as well as for younger adults the PSEs for the chosen and unsorted confidence sets did not deviate from each other (older adults: *t*(28) = 0.06, *p* = 0.953, *d* < 0.01; younger adults: *t*(29) = − 0.05, *p* = 0.960, *d* < 0.01).

### Confidence efficiency

In order to investigate individual differences in metacognitive efficiency, we analysed the sensitivity increase for the set of perceptual decisions chosen as confident relative to the set of unsorted decisions as a percentage of the sensitivity derived from the unsorted decisions, i.e., the CMI (see “Methods”). Figure 5c gives these confidence efficiencies. We initially used one-sample *t*-tests to evaluate whether CMIs differed from zero. Results supported positive CMIs in older adults, *t*(28) = 8.21, *p* < 0.001, *d* = 1.52, as well as in younger adults, *t*(29) = 18.99, *p* < 0.001, *d* = 3.47. Both age groups thus showed some ability to judge the validity of their perceptual decisions. However, on average, metacognitive sensitivity was significantly lower in older compared to younger adults, *t*(45.34) = − 2.51, *p* = 0.016, *d* = − 0.66. Whereas the link between confidence judgements and objective accuracy of perceptual decisions triggers a relative sensitivity benefit of over 30% in younger adults, *M* = 31.21 ± 1.64, the benefit is limited to less than 25% in older adults, *M* = 23.04 ± 2.81. Please note that we observed substantial variability of CMIs in our sample, especially pronounced in the group of older adults (Levene’s test: *F* = 4.87, *p* = 0.031). We next aimed to scrutinize which functional capacities drive the described age effect.

**Figure 6:**
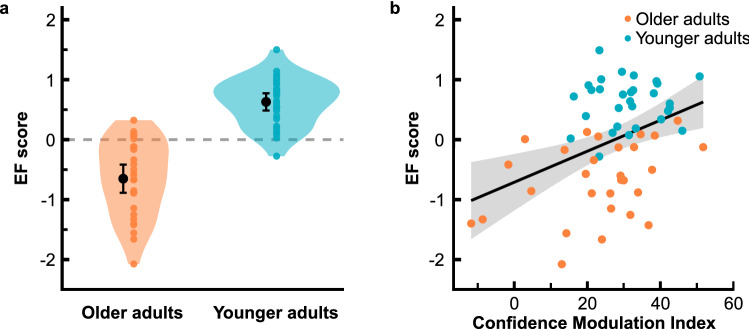
Cognitive control capacities and metacognitive sensitivity. (**a**) EF score as a function of age group; EF scores provide a combined measure for cognitive control capacities averaging *z*-scores from DSST, TMT-B, VST-C, and LPS-3; coloured dots illustrate individual data and black dots represent the mean; shaded areas display 95% of the data distribution smoothed by a kernel density function. Error bars give 95% confidence intervals. (**b**) EF scores as a function of CMIs; data for older and younger adults are plotted in different colours; shaded area gives 95% confidence interval.

We were particularly interested in the role of cognitive control capacities since their decline essentially characterizes cognitive ageing. We captured them by an EF score covering key facets. Figure 6a gives EF scores in both age groups. On average, older adults showed less cognitive control capacities than younger adults, *t*(46.88) = − 9.37, *p* < 0.001, *d* = − 2.44.

We investigated the link between confidence efficiency and cognitive control capacities considering our complete sample in order to comprehensively exploit interindividual variability. Figure 6b illustrates the link between the CMI and the EF score. We determined a robust correlation of *r*(59) = 0.40, *p* = 0.001, 95% CI [0.17, 0.57]. EF scores explained 16% of the variance in confidence efficiency. Depiction of age group membership for each data point suggests that this correlation is not merely driven by group differences but actually describes a general link. Consistently, a partial correlation analysis controlling for the factor age group, though attenuating the correlation, yielded corresponding results, *r*(56) = 0.26, *p* = 0.045. 95% CI [− 0.01, 0.50]. Our findings thus indicate that age-related differences in metacognitive efficiency are crucially driven by cognitive control capacities.

Short-term memory capacity represents another resource that is subject to prominent age-related changes. Considering that the procedure of our paradigm putatively necessitates relevant memory resources, we wanted to check whether the age effect on confidence efficiency can be explained by a confound inherent to the task demands. The digit span measure we used to assess short-term memory capacity indicated significantly lower capacities in our older adult group, *t*(57) = − 2.82, *p* = 0.007, *d* = − 0.58. However, we found no evidence that the CMI is linked to individual differences in memory capacity, *r*(59) = 0.12, *p* = 0.363, 95% CI [− 0.17, 0.41]. Given this result, we consider it as rather unlikely that confidence efficiency had been compromised by task demands that might be more challenging for older adults with lower memory resources.

**Figure 7:**
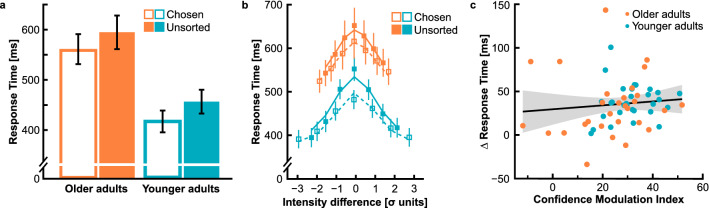
Response times (RT). (**a**) Average RTs as a function of age group and confidence set; open bars illustrate data from the chosen confidence set, closed bars represent data from the unsorted confidence set. (**b**) RTs for bins of stimulus intensities, i.e., contrast difference between test and standard patches, given in standard deviation units of the psychometric function (see “Methods”); symbols represent average group data, lines represent the average fitted data; dashed lines and open symbols represent data from the chosen trial set, solid lines and closed symbols represent data from the unsorted trial set; colour code for age groups corresponds to (**a**). (**c**) RT differences between the unsorted and the chosen confidence sets as a function of CMIs; data for older and younger adults are plotted in different colours. Error bars and shaded areas give 95% confidence intervals.

We finally explored whether age-related slowing could contribute to differences in metacognitive efficiency. Since confidence scales with response times, i.e., higher confidence is linked to faster responses, lower confidence to slower responses, the calibration of confidence judgements might critically rely on timing dynamics. Increased processing time might be detrimental to metacognitive efficiency. First, we analysed median RTs by a two-factorial ANOVA with *age group* as between-subjects factor and repeated measures on the factor *confidence set*. Figure 7a shows average RTs as a function of age group and confidence set.

We observed a significant main effect for *age group*, *F*(1, 57) = 13.38, *p* = 0.001, η_p_^2^ = 0.19, indicating slower RTs for older adults (chosen: *M* = 561 ± 30 ms; unsorted: *M* = 595 ± 33 ms) as opposed to younger adults (chosen: *M* = 419 ± 21 ms; unsorted: *M* = 457 ± 23 ms). In addition, a significant main effect of *confidence set* supported faster RTs for the chosen confidence set, *F*(1, 57) = 88.17, *p* < 0.001, η_p_^2^ = 0.61. There was no interaction between both main effects, *F*(1, 57) = 0.32, *p* = 0.572, η_p_^2^ < 0.01. The relationship between RTs and confidence was similar in both age groups.

Since RTs are not only affected by confidence but also by stimulus difficulty, we further clarified potential age-specific contributions. We disentangled both factors by modelling the RTs in each age group with three free parameters (see “Methods”). Fitting results are illustrated in Fig. 7b. Consistent with the previous analysis, the first parameter α, giving the generic RT, significantly differed between the two age groups (older adults: *M* = 524 ± 31 ms; younger adults: *M* = 438 ± 25), corroborating age-related slowing, *t*(57) = 2.23, *p* = 0.030, *d* = 0.58. For both parameters β and γ, giving the influence of stimulus intensity and confidence on RTs, respectively, we determined values that consistently differed from zero for older and younger adults (all *p*’s < 0.001). RTs became slower with decreasing stimulus intensity, i.e., increasing difficulty, and faster with confidence. Most importantly, neither the parameter β nor the parameter γ differed between age groups (β: *t*(33.17) = − 0.43, *p* = 0.672, *d* = -0.11; γ: *t*(57) = − 0.71, *p* = 0.483, *d* = -0.18). These results corroborate that perceptual decision times underlie similar mechanisms in older and younger adults. Concluding, we directly tested whether the RT differences in the chosen relative to the unsorted confidence set were linked to confidence efficiency. Figure 7c gives the RT differences as a function of the CMI. Both parameters were not significantly correlated, *r*(59) = 0.10, *p* = 0.450, 95% CI [− 0.18, 0.44]. Overall, RT analyses suggest that individual differences in metacognitive efficiency do not emerge from processing speed dynamics.

## Discussion

Our perception relies on decisions about sensory evidence and the subjective confidence in the accuracy of these decisions. Visual perception is subject to pronounced age-related changes, however, the complexity of processes that contribute to these changes is still not well understood^7^. In this study, we were interested in age effects on visual confidence, i.e., the ability to evaluate the quality of one’s own perceptual decisions. Given age-related vulnerabilities in neural and cognitive resources that have been shown to be critical for metacognition, we hypothesized that confidence efficiency decreases with age.

We investigated visual confidence in a sample of healthy older and younger adults with an established confidence forced-choice paradigm that avoids idiosyncratic judgement biases^71,72^. We characterized participants’ executive function capacities using a comprehensive executive function (EF) score that covers the key facets of cognitive control. We were thus able to examine the role of individual differences in cognitive control resources for confidence efficiency. Our results show that older adults do have access to a reliable measure of their uncertainty underlying perceptual decisions. Confidence judgements were consistently linked to the accuracy of perceptual decisions in both age groups. However, the efficiency of this link significantly decreases with age. While confidence judgements explained a sensitivity benefit of over 30% in younger adults, this benefit was limited to less than 25% in older adults. Across our participants we observed substantial individual differences in confidence efficiency. We determined that 16% of the variance in confidence efficiency can be explained by individual cognitive control resources. Importantly, the critical impact of executive function was not exclusively defined by age-related differences, but showed as a general functional link that drives individual differences in metacognition.

Our findings provide critical evidence for age-related differences in metacognition across the adult lifespan and expand our understanding on how it impacts visual perception. In the confidence forced-choice paradigm, we observed that older adults could selectively choose the interval that led to a higher performance in some cases. This indicates that they can evaluate the quality of their percepts. When compared to younger adults, though, this ability is reduced on average. Since our paradigm was tailored to minimize the impact of response and confidence biases that have been shown to vary with age^29,32^, our results support original age effects on metacognition in a visual task. Congruently, the only previous study concerned with such effects reported reduced performance introspection with increasing age^28^. However, those findings remained ambiguous. Older adults showed lower awareness of their perceptual performance, but confidence was assessed by ratings scales which might make the evaluation of confidence sensitivity prone to confounding biases^42,43^. In addition, inconsistent results across different functional domains that emerged in the study await further clarification^49–52^.

Our findings might be complicated by several factors that require careful consideration. Task difficulty might affect quality of confidence judgements. For our contrast discrimination task, we chose sinusoidal gratings with a spatial frequency of 0.8 cyc/° for which age differences in contrast sensitivity were expected to be negligible^76^. We yet found clear age effects on contrast discrimination thresholds, putatively triggered by relatively short presentation times^77,78^. Older adults showed higher thresholds and given that we used the method of constant stimuli for threshold measurement, higher task difficulty is implied for our group of older adults. Differences in task difficulty could, in turn, compromise confidence decisions^79^. Whereas rather difficult tasks compromise identification of high confidence trials, rather easy tasks compromise identification of low confidence trials. However, the fit of the psychometric functions suggested that the applied intensity range was well-suited to capture performance across age groups. There was no difference between the quality of fits in both age groups. Thus, we consider it as rather unlikely that probably unavoidable differences in task difficulty can explain the systematic age effects on the accuracy of confidence judgements. Furthermore, we ruled out that differential task difficulties emerging from short-term memory affordances explain age-related differences in visual confidence. Older and younger adults differed significantly in short-term memory resources, but we could not determine a relevant impact of this parameter on the efficiency measure derived from our paradigm.

It might be also speculated that differences in processing speed can contribute to age effects on visual confidence. The reduction of processing speed is probably the most pronounced and robust functional age difference^11,80^. Higher confidence in perceptual decisions is found to be associated with faster response times^74,75^. This link could basically rely on two mechanisms. First, observers might consider response times when forming their confidence judgements, deriving a higher confidence judgement from shorter response times. Although we assume that confidence judgements were primarily based on an appraisal of accuracy, response times were likely integrated to some degree. Secondly, the implicit experience of confidence at the time of the perceptual decisions might speed up responses. Since the detailed timing of processes that contribute to forming a confidence judgement eludes examination, both processes are difficult to distinguish. However, age-related slowing might critically interfere with both mechanisms and thus could be detrimental to metacognitive efficiency in older adults. As expected, we determined significantly prolonged response times in older adults compared to younger adults. However, and importantly, response times were similarly modulated by confidence in both age groups. We found that, independent of age, responses were speeded up for perceptual decisions that are judged with higher confidence. In sum, we thus corroborate previous results showing differences in response times as a function of confidence in younger adults^74,75^ and extend these findings to older age. Individual differences in processing speed do not interfere with efficient confidence judgements. In contrast, response times are consistently shaped by the confidence in the accuracy of perceptual decisions.

A main focus of our study was on the link between executive function and visual confidence. Given the substantial conceptual overlap between metacognition, i.e., monitoring of decision quality, and executive function, i.e., cognitive control, a functional relationship suggests itself^4,23,44^. Both concepts have been shown to rely on shared neural resources, in particular in the prefrontal cortex^14–16,81,82^. Recent studies suggest that this functional overlap might specifically represent the signature of domain-general processes that characterize metacognition^82,83^. Ageing offers a powerful proxy to individual differences in executive function^11,18,25–27^. For example, critical age-related differences in error monitoring have been described, a capacity that can be plausibly linked to confidence judgements^84,85^. We captured individual cognitive control resources in a comprehensive score of executive function that was supposed to cover facets of the concept broadly^23^. Older adults on average showed lower EF scores than younger adults, consistent with established findings of age effects on executive function^11^. Thus, cognitive control resources could be identified as a plausible candidate driver of age-related differences in metacognitive efficiency. Most importantly, we were able to exploit the variability in EF scores across our older and younger participants to reveal a general functional link between cognitive control resources and visual confidence. Please note that the measures that contribute to our EF score exclusively rely on visual information processing. Although we cannot exclude that this congruency with our perceptual task might contribute to the reported link to some degree, we suggest that a significant impact is rather unlikely. Indeed, almost all established measures of executive function rely on visual information and are considered as indicative for cognitive control capacities across heterogenous tasks^24^. Our finding is in line with previous evidence suggesting that metacognition basically relies on cognitive control resources^33,86,87^. We are aware of conflicting results indicating that metacognition and cognitive control might be better understood as independent capacities^28,40^. However, we suggest that in some studies the functional links might be attenuated by executive function measures covering only specific facets of the concept. In addition, restriction of the range of individual differences in cognitive control resources due to very homogenous samples with regard to age and education can be assumed to obscure functional links.

Our study was dedicated to visual confidence; thus, we can only speculate whether our findings also hold for confidence in other perceptual domains and even more generally for other functional domains, in particular metamemory. Although behavioural evidence suggests some domain-specific contributions to metacognition^82^, overall general, domain-independent mechanisms are proposed and supported by neuroimaging^50,82,83,87,88^. For confidence in perceptual tasks, findings consistently suggest similar mechanisms across different tasks and modalities^74,75,82^. Heterogeneity of results with regard to age effects on metamemory hampers systematic evaluations^31,33,35,38^. Inconsistent results might emerge in part from specific biases due to applied methods of measuring metacognitive parameters. In summary, we propose that our findings on age effects and the pivotal impact of cognitive control resources hold not only for visual confidence but also for confidence in other perceptual domains and more generally for other decision tasks.

To conclude, we showed that older adults have access to a reliable measure of their own uncertainty when making visual decisions. Metacognitive capacities are key for behavioural control. For instance, a reduced performance introspection could result in not being able to identify relevant aspects of a task and inefficient allocation of resources^89^. However, we found clear age-related differences in metacognition. Our results suggest reduced confidence efficiency in older adults. In principle, these age effects could be due to compromised reliability of judgements but also due to declining cognitive control resources^90^. Exploiting individual differences across our complete sample, we corroborated the crucial functional role of cognitive control resources for metacognition. We propose that age effects on visual confidence are primarily mediated by this functional link. This finding is in line with converging evidence that age-related changes in perception and sensorimotor control are critically driven by executive contributions to efficient resource control^91–94^.

## Data Availability

Data are publicly available at the 10.5281/zenodo.5257748.
